# Quality of life and self-stigma of schizophrenia patient’s caregiver tool: Development and validation using classical test theory and Rasch analysis

**DOI:** 10.4102/sajpsychiatry.v27i0.1656

**Published:** 2021-11-30

**Authors:** Zainab Albikawi, Mohammad Abuadas

**Affiliations:** 1 Community and Psychiatric/Mental Health Nursing Department, Nursing College, King Khalid University, Khamis Mushait, Saudi Arabia

**Keywords:** schizophrenia, caregivers, validity, reliability, Rash analysis, quality of life, self-stigma, psychological well-being

## Abstract

**Background:**

Providing care for schizophrenia patients is complex, and it requires dealing with various psychosocial burdens.

**Aim:**

To develop and validate a tool that measures the quality of life and self-stigma (SS) of the schizophrenia patient’s caregiver (QLSSoSPC).

**Setting:**

Outpatient psychiatric services clinics in Saudi Arabia.

**Methods:**

The current study used a methodological cross-sectional design. A sample of 205 schizophrenia patients’ caregivers was recruited by using a convenient sampling method. Classical Test Theory and Rasch Analysis approaches were used.

**Results:**

The developed tool has proven acceptable level of reliability and validity. The analysis confirmed seven-factor structure accounted for 74.4% of the total variance. Cronbach’s reliability statistics for the developed tool were satisfactory and ranged from 0.80 to 0.91.

**Conclusion:**

The psychometric properties of the QLSSoSPC tool supported its prospective use and allowing us to recommend the implementation of the tool on behalf of clinical and research purposes.

## Introduction

Schizophrenia is a complex and multi-causal syndrome. Its prevalence is about 1.5 per 10 000 people.^1^ Age of onset is usually during adolescence; childhood and late-life onset is rare. Slightly more males are diagnosed with schizophrenia than females (1.4:1),^2^ and females tend to be diagnosed later in life than males.

Schizophrenia is considered a serious psychological disorder that influences the lives of patients and patients’ caregivers. Caregivers’ responses regarding having a family member with schizophrenia disorder are care burden, shame about illness, uncertainty about the disease and stigma.^3^ The responsibility of caregivers has gained a major function, as a consequence of increasing shift of psychiatric healthcare facilities to the community. Psychiatric patients received moderately short inpatient healthcare and early discharge from the healthcare institution adds to the important role of caregivers.^4^

A caregiver is defined as a family individual who is existing for more than 1 year with the patient and has directly participated in patient’s daily living activities, social interaction and psychiatric healthcare issues.^5^ Schizophrenia also influences the performance and health of family caregivers, mostly because they have assumed the responsibilities that were performed previously via psychiatric healthcare institutions.^6^

The life-quality of family caregivers is greatly affected by the care provided to the schizophrenia patient.^7^ Schizophrenia patient’s caregivers have been observed to have low quality of life, particularly when experiencing a significant burden.^8^ In addition, caregivers’ depressing experience may influence their capability to take care of their patients.^9,10^ Recent evidence emphasised that improving quality of life has significant impact on the health of both the caregivers and their patients’. In spite of the wealth of literature that addressed specific issues related to care giving, little has been conducted to explore quality of life amongst schizophrenia patient’s caregivers. Moreover, there is few studies that developed a tool to measures the life-quality of the schizophrenia patient’s caregiver.^7,8^

Providing care for schizophrenia patients is complex and technical, and it requires dealing with various psychosocial burdens. Family members often assume the care giving role with little or no preparation and without considering whether they have enough psychological and social preparations.^11,12^. Stigma is one of the main demanding psychosocial burdens experienced by the caregivers of the mentally ill patients.^13,14,15,16^ A large study of family caregivers in Morocco found that caregivers experienced a high level of perceived stigma.^17^

Consequently, care-giving (i.e. providing care to patients with schizophrenia) has a substantial impact on family caregivers’ social and mental well-being, and can negatively influence patient and caregiver health outcomes.^14^ Other researchers found a significant association amongst caregivers’ self-stigma, psychological stress and quality of life.^18,19,20^ A recent study found a negative significant relationship between self-stigma and quality of life, where those who had higher self-stigma were more expected to have poorer quality of life.^21^

There is a strong consensus supporting that caring for an individual with schizophrenia is burdensome and stressful to family caregivers and contributes to significant reduction in quality of life for family members. Studies agreed with the consideration that quality of life, especially of family caregivers of schizophrenia patients, is mainly linked to many essential factors, that include: (1) individual well-being, (2) psychological status, (3) functional abilities, (4) social interaction, (5) occupational status, (6) financial status and (7) physical health (PH).^22^ As these studies indicate, it is essential to have well-prepared caregivers and to promote their quality of life because caregivers play a critical role in helping patients deal with the effects of mental illness and its treatment or maintain their health.

To provide an understanding of the quality of life for family caregivers of patients with schizophrenia and their need for better preparation and psychosocial care, this state-of-the-science examines the most essential domains of quality of life for family caregivers. Therefore, there is a need to develop and validate a tool for evaluating quality of life and self-stigma (SS) for the schizophrenia patient’s caregiver. Up to now, reliable and valid tool that clearly and sufficiently incorporates the concept of SS; besides the main domains of quality of life have not been available, that’s why the quality of life and SS of the schizophrenia patient’s caregiver (QLSSoSPC) represent a significant new development. The current study aimed to develop and validate a tool that measures the QLSSoSPC.

## Methods

### Study design

The current study used a methodological cross-sectional design.^23^

### Sample and data collection

Sample size was considered in line with the recommendations of Nunnally^24^ who advised a graded scale to determine sufficient sample size for tool development as the following: poor = 100, fair = 200, good = 300. Out of 310 participants who were selected by convenience sampling methods and were asked to join the study, a sample of only 205 participants who matched the study inclusion criteria participated in the existing study. The inclusion criteria were: (1) being the father, mother, sisters or brothers of patients with schizophrenia, (2) schizophrenia patients being diagnosed based on *Diagnostic and Statistical Manual of Mental Disorders* (DSM-IV) criteria, (3) caregivers’ age of 18 years old or more and (4) being able to speak and read Arabic language.

Data were collected and the participants were recruited from two psychiatric outpatient’s clinics in Saudi Arabia. Participants were approached and screened for eligibility by: (1) accessing their medical health files, (2) identification of first-degree relative caregivers who take care of schizophrenia patients. The researchers invited and distributed the questionnaires to the eligible caregivers. Consent forms and cover letters were enclosed with the questionnaires. The researcher included full details of the study, information that participation was anonymous and voluntary and contact information for the researchers. The current study was approved by the Research Ethics Committee of the institution.

### Measures

The study data were collected through the use of the following tools. The first tool was used to collect the socio-demographic characteristics of the caregivers (gender, age, number of family members and occupation). The second tool was used to collect the clinical information about schizophrenia patients (type of schizophrenia, age at diagnosis, duration of schizophrenia). The third tool was the Arabic version of the short-form of the World Health Organization’s quality of life (WHOQOL-BREF 1998).^25^ The WHOQOL-BREF contains 26-items that cover four domains: physical Health (PH, 7 items), psychological (Ps, 6 items), social relationships (SR, 3 items) and environment (E, 8 items). As no cut-points exist to categorise quality of life measured by WHOQOL-BREF, the final score ranges from 0 to 100. The fourth one is a researcher-developed QLSSoSPC.

### Tool development procedure

The final draft of the QLSSoSPC was developed in two stages: (1) qualitative stage in which items were generated, and (2) quantitative stage in which items were reduced and the QLSSoSPC was validated.^26^ The tool was originally developed in Arabic language since all participants were from Saudi Arabia. The study participants were different in the two stages.

#### Qualitative stage

A focus group design was used to investigate the family caregivers’ view and experiences about their quality of life when caring of schizophrenia patients. A focus group method is particularly suitable for obtaining information from several perspectives.^23^ According to Morgado and Meireles,^27^ the qualitative information, derived from direct participants’ observations such as participants’ interviews, can be used to recognise the domain items.

The participants from which the sample was drawn consisted of 15 caregivers who were arranged in three groups. Initially, the authors primarily employed information from the literature review and pre-existing scales to generate discussion questions that reflect the main domains related to the quality of life and self-stigma. The topics developed included 12 open-ended questions that were related to main domains. These questions were used to stimulate discussion. Those focus group discussions focused on quality of life and self-stigma as perceived by family caregivers who are caring of patients with schizophrenia. The authors used those discussions to establish the wording in question stems and the range of answer choices. All focus group discussions were recorded and thoroughly transcribed. The qualitative content analysis led to the emergence of 57-items from the focus group data.

After formulating the initial draft of items, the 15-caregivers were asked to give comments on any part of the tool such as: response options, content and tool items wording that they felt was unrelated or needs any improvement. Study items that were ambiguous, misunderstood were withdrawn or reworded that resulted in development of a preliminary tool that comprised 51 items. Finally, the focus group discussions guaranteed that the tool is the reflection of their experiences as the schizophrenia patient’s caregiver.

#### Quantitative stage

**Content validity:** A panel of experts was invited to be involved in the content validity assessment. Six experts in psychiatric and mental health field evaluated the content validity of the first version of QLSSoSPC tool in terms of its relevance, clarity, meaningfulness and completeness using a two-step review process. In the first step, the list of all items was given to the experts, to evaluate and modify these potential items based on the following two criteria: (1) were the items suitable and consistent with the domain? and (2) was the phrasing of the items concise and accurate?

According to the suggestions made by the experts, the first version of the tool was reduced to 30-items. In the second step, the revised draft of questionnaire was judged for relevance using a 4-point ordinal scale (1 – not relevant, 2 – somewhat relevant, 3 – quite relevant, 4 – highly relevant). The panel of experts was selected carefully and had the following credentials: (1) two experts were full professors of psychiatric and mental health nursing, two were clinician experts in psychiatric and mental health and the others were assistant professors of community health nursing; and (2) all experts had numerous publications related to psychiatric, mental health and community care. The following criteria were used to establish the content validity: (1) Content Validity Index-Item (I-CVIs) > 0.78, and (2) Content Validity Index-Scale (S-CVI) > 0.90. The results of the I-CVI confirmed that 83% (*n* = 25) of the items were scored as acceptable (values ranged from 0.83 to 1.00). I-CVIs of 5 items of the 30 items did not meet the cut-off of 0.78. These five items were removed from the tool, resulting in a 25-items version. The content validity of the overall tool was established based on the S-CVI score which was 0.92.

#### Creation of a pilot version of the questionnaire

A group of 20 caregivers, whose characteristics matching the inclusion criteria, participated in the pilot testing to evaluate the comprehensibility and feasibility during administration. The time needed to complete the tool was 10–15 min. No further changes in structure or content were in order. Subsequently, the pilot draft of the tool was composted of 25 items written in Arabic covering the seven domains, as follows: Psychological Well-being – PW (5 items); Self-Stigma – SS (7 items); Relationships with Family and Relatives – RFR (3 items); Relationships with Psychiatric Health Team – RPHT (3 items); Physical Health – PH (2 items); Psychological Burden – PB (2 items) and Financial Burden – FB (3 items). A Likert-type format was used with 5-points, ranging from 1 (strongly disagree) to 5 (strongly agree).

Construct validity, reliability and some aspects of external validity were tested for QLSSoSPC tool.

Ceiling and floor effects were considered to evaluate the skewness of the answer distribution. The floor effect reflects the proportion of participants who have the worst possible total score for the tool, as well as the ceiling effect reflects the proportion with the best score that is possible for the tool. A high ceiling or floor effect limits the result of a tool for the reason that persons falling within the highest and lowest averages cannot obtain better or worse scores.^28^ Accordingly, it results in an absolute lack of tool sensitivity.

### Data analysis

Statistical Package for Social Sciences (SPSS) software (Version 21.0) and WINSTEPS software 3.72.3 based on the Rasch model were used to assess the tool items’ appropriateness and suitability.^29^

### Ethical considerations

The current study was approved by the Research Ethics Committee of King Khalid University with approval number: (ECM#2019-57)-( HAPO-06-B-001).

## Results

### Socio-demographic and clinical characteristics

A total of 205 family caregivers used the self-administered tools with a response rate of 81%. The sample mean age was 55 years with a standard deviation of 9.4 years and ranged between 30 and 65 years. A total of 33% were male caregivers, whereas 67% were female caregivers. Nearly half of the study participants had a job (58.5%, *n* = 120). The majority of the sample had more than three members in their family (85.9%, *n* = 176). For patients with schizophrenia, the mean of age since diagnosis was 22.3 years with a standard deviation of 6.7 years (see Table 1).

**TABLE 1:** Caregiver’s socio-demographic characteristics and clinical information (*n* = 205).

Variables	*N*	%
**The caregivers**		
Mean of age (years) ± s.d.	55	9.4
Gender (Male)	68	33.2
Caregivers’ family members ≥ 3	176	85.9
Have Occupation	120	58.5
**The patients with schizophrenia**		
Schizophrenia duration (years) ± s.d.	8.7	9.6
Age (mean) at diagnosis (years) ± s.d.	22.3	6.7
**Types of schizophrenia**		
Undifferentiated	27	13
Disorganised	9	4.3
Residual	40	19.4
Schizoaffective	10	5.1
Paranoid	119	58.2

s.d., standard deviation.

### Scoring

For each caregiver, a score for each tool domain was obtained by calculating the mean of the tool item scores on the domain. But if less than half of the QLSSoSPC items were missing, non-missing items mean was given for the missing items. The scores of all domains were linearly standardised to a 0–100 scale, with 100 score indicating the highest level of quality of life and 0 the lowest.

### Construct validity

Item iteration process confirmed the hypothesised seven-dimension factor structure with 25-items. The scalability was considered acceptable and all items showed a good fit to the Rasch model within each dimension, with no items showing an item goodness-of-fit statistics (INFIT) outside of the acceptable range (value between 0.7 and 1.2). Also, all items on each factor were from the same domain, and the total explained variance by all factors together was 75.6%. For each domain, name was given according to its items, as the followings: PW (5 items); SS (7 items); RFR (3 items); RPHT (3 items); PH(2 items); PB (2 items) and FB (3 items) (see Appendix 1). The results established an absence of DIF related to participants’ age and gender which supported the invariance of the tool item calibrations.

### Reliability

The reliability was determined using internal consistency methods. The internal consistency for the whole sample and each subscale was determined by Cronbach’s α. Cronbach’s α of about 0.7 is considered sufficient. Furthermore, the following criteria were applied to recognise poorly functioning items: (1) a correlation of less than 0.30 between an item and the subscale score or (b) an increase of > 0.10 in the total questionnaire reliability when the item was deleted.^30^

Cronbach’s α ranged from 0.80 and 0.91 in the whole sample. All the corrected item–total correlations were found to be > 0.30, indicating high internal consistency. The deletion of any of the 25 items did not increase the internal consistency of any of the seven dimensions. Item internal consistency was satisfactory for all dimensions ranging from 0.63 to 0.94 for each item. The correlation of each item with its associated dimension was higher than its correlation with the other dimensions (item discriminant validity) (See Table 2).

**TABLE 2:** Characteristics of tool domains (*n* = 205).

Domain	PW	SS	RFR	RPHT	PH	PB	FB	Index
**Mean**	52.8	61.7	68.4	56.8	61.8	68.1	61.1	-
s.d.	17.4	15.3	20.8	21.7	19.6	21.3	10.3	-
**Item-internal consistency**								
Min-Max	0.82–0.91	0.63–0.77	0.73–0.90	0.91–0.94	0.92–0.93	0.92–0.93	0.79–0.87	N/A
**Item discriminant validity**								
Min-Max	0.01–0.57	0.00–0.50	0.00–0.38	0.00–0.28	0.00–0.40	0.05–0.33	0.06–0.41	N/A
**Missing values**								
%MV	0.3	0.3	7.6	5.2	4.6	4.8	5.2	18.9
**Floor effect**								
%	10.6	12.1	7.6	7.1	16.5	7.7	10.1	10.3
**Ceiling effect**								
%	6.0	13.8	44.3	10.8	9.7	10.1	32.6	15.4
Cronbach’s alpha	0.91	0.87	0.81	0.91	0.86	0.87	0.80	0.86
**Rasch statistics (INFIT)**								
Min-Max	0.71–1.10	0.78–1.17	0.61–1.20	0.75–1.12	0.95–0.98	0.97–0.98	0.77–1.17	N/A

PW, psychological well-being; SS, self-stigma; RFR, relationships with family and relatives; RPHT, relationships with psychiatric health team; PH, physical health; PB, psychological burden; FB, financial burden; N/A, not applicable; INFIT: item goodness-of-fit statistics, % MV: missing values, s.d., standard deviation.

### External validity

The QLSSoSPC index was significantly correlated with all WHOQOL-BREF domains scores (rs = 0.18–0.43). However, only two domains of the QLSSoSPC (PW, SS) showed medium to high correlations with domain scores of the WHOQOL-BREF assessing PW and SS: PW-PH, PW-Ps, PW-SR, PW-E and SS-Ps. On the contrary, the specific domains of the QLSSoSPC: PH, PB and FB were uncorrelated or weakly correlated with the domains of WHOQOL-BREF (See Table 3).

**TABLE 3:** Spearman’s correlations between quality of life and self-stigma of schizophrenia patient’s caregiver and WHOQOL-BREF scores (*n* = 205).

Domain	PW	SS	RFR	RPHT	PH	PB	FB	Index
PH	0.45**	0.26**	0.13*	0.10	0.17**	0.12	0.14*	0.36**
Ps	0.52**	0.40**	0.12*	0.10	0.22**	0.10	0.06	0.36**
SR	0.42**	0.27**	0.14*	0.05	0.17**	0.13*	0.12*	0.32**
E	0.36**	0.24**	0.15	-0.06	0.07	0.05	-0.00	0.16*

WHOQOL-BREF, the Arabic version of the Short-form of the World Health Organization’s quality of life; PW, psychological well-being; SS, self-stigma; RFR, relationships with family and relatives; RPHT, relationships with psychiatric health team; PH, physical health; PB, psychological burden; FB, financial burden; PH, physical health; Ps, psychological; SR, social relationships; E, environment.

* , *p* < 0.05;

** , *p* < 0.01.

In comparison between male and female participants, results showed significant differences for the following two domains: PW for male was 58.2 (18.3) and female 50.3 (16.5), *p* = 0.003; and RFR for male was 76.6 (16.0) and female 64.2 (24.0), *p* < 0.0001. Family consists of three and more members, who were associated with a higher tool score for FB and the result of index. Caregivers without job presented a significant high level for RFR as well as a lower level of PB. Moreover, caregivers of patients with paranoid schizophrenia type showed lower tool scores (RPHT and the index). And the highest total scores for RPHT and the index are shown in Table 4.

**TABLE 4:** The quality of life and self-stigma of schizophrenia patient’s caregiver tool domains scores means (standard deviation) according to socio-demographic characteristics and clinical information (*n* = 205).

Variables	PW			SS			RFR			RPHT			PH			PB			FB			Index		
	Mean	s.d.	*p*	Mean	s.d.	*p*	Mean	s.d	*p*	Mean	s.d	*p*	Mean	s.d	*p*	Mean	s.d	*p*	Mean	s.d	*p*	Mean	s.d	*p*
**Gender**	-	-	0.003*	-	-	0.063	-	-	< 0.0001*	-	-	0.068	-	-	0.176	-	-	0.211	-	-	0.670	-	-	0.125
Male caregivers	58.2	18.3	-	65.5	17.3	-	76.6	16.0	-	58.5	20.6	-	61.7	21.8	-	61.6	22.0	-	71.2	20.6	-	63.2	11.3	-
Female caregivers	50.3	16.5	-	60.4	14.7	-	64.2	24.0	-	63.6	21.2	-	55.4	22.5	-	63.2	21.5	-	67.6	21.7	-	60.8	11.7	-
**Caregivers’ family members**	-	-	0.429	-	-	0.202	-	-	0.062	-	-	0.084	-	-	0.742	-	-	0.221	-	-	0.020*	-	-	0.032*
< 3	52.6	17.4	-	58.2	18.5	-	62.6	20.1	-	57.4	21.1	-	56.2	20.7	-	57.8	21.7	-	62.8	17.6	-	57.2	8.7	-
≥ 3	53.2	17.6	-	62.4	14.5	-	71.7	20.8	-	64.0	20.5	-	57.2	24.0	-	62.5	21.7	-	71.6	21.7	-	62.6	12.4	-
**Occupation**	-	-	0.684	-	-	0.262	-	-	0.013*	-	-	0.162	-	-	0.787	-	-	0.012*	-	-	0.622	-	-	0.527
Working	53.5	18.2	-	60.5	15.7	-	64.0	21.6	-	64.4	21.0	-	57.1	21.4	-	66.3	21.3	-	68.7	23.6	-	62.3	10.4	-
Not working	52.5	17.2	-	62.5	15.1	-	71.1	22.1	-	61.6	21.1	-	56.7	22.1	-	61.2	21.6	-	67.6	21.3	-	61.2	12.4	-
**Types of schizophrenia**	-	-	0.123	-	-	0.074	-	-	0.225	-	-	0.012*		-	0.102	-	-	0.517	-	-	0.246	-	-	0.007*
Undifferentiated	55.1	17.4	-	64.5	14.3	-	63.3	23.5	-	65.0	22.7	-	58.3	22.7	-	60.1	21.7	-	71.3	21.3	-	61.4	8.6	-
Disorganised	45.4	6.6	-	62.7	6.8	-	82.2	14.2	-	81.0	16.7	-	78.6	17.2	-	71.2	22.3	-	81.7	13.2	-	73.6	7.2	-
Residual	58.6	21.3	-	66.0	16.7	-	71.2	23.2	-	62.1	18.6	-	61.6	23.7	-	61.5	20.5	-	72.0	18.5	-	64.5	11.3	-
Schizoaffective	51.7	16.5	-	68.24	16.3	-	72.1	26.2	-	77.6	17.5	-	61.4	30.7	-	71.1	15.4	-	71.6	22.3	-	67.3	13.7	-
Paranoid	51.2	17.1	-	61.5	17.6	-	67.6	21.6	-	62.5	21.5	-	55.0	23.2	-	61.3	22.1	-	66.0	22.5	-	62.0	11.7	-

PW, psychological well-being; SS, self-stigma; RFR, relationships with family and relatives; RPHT, relationships with psychiatric health team; PH, physical health; PB, psychological burden; FB, financial burden; s.d., standard deviation.

* , *p* < 0.05.

The SS, RFR and PB domains scores significantly correlated with the study caregiver’s age 0.23, 0.13 and -0.24, respectively. The patient age at diagnosis of schizophrenia had also significant correlation rs = (0.13–0.23) with SS, RFR, PH, FB and the index (See Table 5).

**TABLE 5:** Spearman correlation of the total score of the quality of life and self-stigma of the schizophrenia patient’s caregiver tool with caregiver’s age and patient’s age at diagnosis.

Variables	PW	SS	RFR	RPHT	PH	PB	FB	Index
Caregiver’s age	0.11	0.23**	0.13*	-0.02	-0.06	-0.24**	0.08	0.02
Patient age at diagnosis	0.06	0.17*	0.13*	0.05	0.17**	0.07	0.16*	0.23**

PW, psychological well-being; SS, self-stigma; RFR, relationships with family and relatives; RPHT, relationships with psychiatric health team; PH, physical health; PB, psychological burden; FB, financial burden.

* , *p* < 0.05 and

** , *p* < 0.01.

## Discussion

The main aims of this study were to develop and validate a tool designed to assess the QLSSoSPC. Developing and validating a new tool to assess multiple important domains related to QLSSoSPC arose from the fact that most of the available tools were not addressing self-stigma as an influencing factor.^31^ Thus, providing a reliable and valid tool for the schizophrenia caregivers is needed. The QLSSoSPC assesses significant factors that must be discussed when planning care for patients with schizophrenia. Neong and Rashid^31^ recommended that the healthcare givers and policymakers recognise the factors that affect the caregivers quality of life and SS, bearing in mind that more patients with psychiatric health disorders are being cared for in the patients’ community.

Furthermore, SS is considered as the psychological health issue that can be experienced by mentally ill patient’s caregivers, and it is found that caregivers’ SS negatively affects patients’ adherence, as well as treatment seeking and rehabilitation.^32^ Mak and Cheung^33^ studied the relationship between the SS and quality of life amongst mental disorders patients’ caregivers, who found that SS was correlated to caregivers’ quality of life. The current available tools for schizophrenia caregivers are essentially based only on the experts’ or clinicians’ viewpoint, except for the tools developed by Szmukler, Burgess^34^ and Richieri, Boyer.^35^ The components of the QLSSoSPC integrate factors of great significance to patients and are considerably different from other quality of life tools.

Correspondingly, some factors of the QLSSoSPC are similar to those referred to in the literature and the existing tools, such as PW, RFR, RPHT, PH, PB and FB. However, some dimensions, like SS, emerged as a unique concern for the schizophrenia caregivers. The study’s findings indicate that the QLSSoSPC tool is a reliable and valid tool for assessing the caregivers’ quality of life and SS. All the subscales of the QLSSoSPC were internally consistent and mutually exclusive. The Cronbach’s α-coefficients for all items ranged from 0.80 to 0.9, indicating excellent levels of internal consistency.

The findings obtained from Rasch analysis identified seven domains for the QLSSoSPC tool. The majority of the items loaded on their relevant domain, thus confirming the construct validity of the QLSSoSPC tool. Furthermore, the findings obtained indicated that the 25-items of the QLSSoSPC tool fit the data significantly and support the proposed structure. Thus, strong statistical evidence was available to support the construct validity of the seven domains. The psychometric properties for the QLSSoSPC were similar to a few scales developed to assess various sets of factors related to quality of life for schizophrenia caregivers.^34,35^ The development procedure used in the current study was guided by recommendations made by Morgado and Meireles.^27^

An interesting finding from this study was the positive relationship between caregiver’s quality of life and the caregiver’s age for SS and PB domains. Zhai and Du^36^ demonstrated similar results that SS is significantly higher for age 35 years or younger. Moreover, it is found that younger participants’ age was significantly associated with depressive symptoms.^37^ We found that male caregivers had better quality of life than female caregivers, particularly for the domains of PW and RFR. However, the majority of the studies found that female caregivers had lower quality of life.^5,7^

We also found that family caregiver’s quality of life was higher for caregivers who have more than three family individuals; this could be attributed to the help and support for caregivers by other family members. This finding converges with Li, Lambert^8^ study in which they found a positive correlation between caregivers’ quality of life and patient’s family members’ numbers. The findings of the current study indicated significant associations between PB and family and relatives domains. This matches with the findings from a cross-sectional study conducted in Indonesia by Boyer and Caqueo-Urízar^5^ who found that employment condition and social support positively affected quality of life.

One more finding was related to patient’s age at diagnosis of schizophrenia. In our study, the higher quality of life of the caregivers was associated with older age of patient diagnosis. Similarly, Li, Lambert^8^ found that patient diagnosis was significantly associated with older age. The suitability of QLSSoSPC is good. There was low level of missing data that were between 0.3% and 7.6% for all domains. The expected average time to use the tool is likely to be nearly 5 min that will enhance its use in psychiatric research and clinical practice.

There were several limitations to the current study. Firstly, the stability of the tool was not measured over time. Secondly, cross-validation studies are required to confirm the factor structure of the QLSSoSPC tool, and to establish the reliability and validity in other populations. Thirdly, the sample may not be representative because of the self-selection of the conveniently selected participants.

## Conclusion

The QLSSoSPC tool is a novel tool that combined in its structure the factor related to SS that was not measured before by any tool. The existing study tool measures quality of life and SS for schizophrenia caregivers. Also, it is not proposed to substitute the available tools. Rather, the QLSSoSPC tool adds remarkable information to psychiatric healthcare and enables researchers to assess the schizophrenia patients’ caregivers’ quality of life and SS by using one tool. It would be crucial to investigate the reproducibility of the existing study results. However, the QLSSoSPC tool psychometric properties are supported by the rigor of the statistical methods used in the existing study. The originality of the QLSSoSPC tool may be valuable in psychiatric practice and research scope.

## Appendix 1

**TABLE 1-A1:** The Arabic and English version of QLSSoSPC tool.

Domain	Arabic	English
-	على مدى الأشهر الست الماضية ، أنت :	For the last 6 months, you
PW: Psychological Well-Being (5 items)	1. لم تشعر بأي مشاعر ايجابية.	1. could not experience positive feelings.
-	2. تجد صعوبة في التعبير عن مشاعرك.	2. have difficulty to express your feelings.
-	3. تجد صعوبة في التركيز والتفكير الجيد.	3. have difficulty to concentrate and think well.
-	4. شعرت بالقلق والتوتر	4. felt worried and anxious.
-	5. شعرت بالاكتئاب وفقدت الدافعية.	5. felt depressed and have lost motivations.
SS: Self-stigma (7 items)	6. تعتقد أن عائلتك لم تحب زيارتك لأن لديك مريض مصاب بالفصام.	6. believe that your family did not like to visit you because you have a patient with schizophrenia.
-	7. يعاملك الآخرون بطريقة مختلفة بسبب وجود مريض مصاب بالفصام في عائلتك.	7. have been treated by others in different ways because of the presence of patients with schizophrenia in your family.
-	8. فقدت أصدقائك بمجرد علمهم أنك مقدم الرعاية لمريض مصاب بالفصام.	8. lost your friends once they knew that you are a caregiver of the patient with schizophrenia.
-	9. شعرت بالحاجة إلى إخفاء أنك تعتني بمريض مصاب بالفصام.	9. felt the need to hide that you are caring for patients with schizophrenia.
-	01. تتجنب الذهاب إلى المناسبات الاجتماعية مع المريض المصاب بالفصام.	10. avoided to go to social events in the company of patients with schizophrenia.
-	11. شعرت بالخجل أو الإحراج لأن عائلتك لديها مريض مصاب بالفصام.	11. felt shamed or embarrassed because your family has patients with schizophrenia.
-	21. شعرت أن الناس ينظرون إليك بنظرة دونية لأنهم يعرفون أن لديك فردًا من العائلة مصابًا بالفصام.	12. felt that people look down on you as they know that you have family members with schizophrenia.
RFR: Relationships with family and relatives (3 items)	31. تلقيت الدعم من عائلتك.	13. are supported by your family
-	41. تلقيت الدعم من أقربائك.	14. are supported by your relatives
-	51. تم فهمك من قبل العائلة والأقارب.	15. have been understood by your family and relatives.
RPHT: Relationships with Psychiatric Health Team (3 items)	61. تم فهمك من من قبل فريق الرعاية الطبية والتمريضية.	16. have been understood by medical and nursing care team.
-	71. حصلت على المساعدة من قبل فريق الرعاية الطبية والتمريضية.	17. have been taking help from medical and nursing care team.
-	81. راضي عن المعلومات والتوضيحات التي يقدمها فريق الرعاية الطبية والتمريضية.	18. are satisfied with the information and clarification given by medical and nursing care team.
PH: Physical health (2 items)	91. شعرت بالارهاق والتعب.	19. felt overloaded and tired.
-	02. لا تستطيع الاسترخاء وتفتقر إلى الطاقة الجسدية.	20. are not able to relax and lack physical energy.
PB: Psychological Burden (2 items)	12. شعرت أنك لست حراً حتى تقوم بأنشطة الحياة اليومية الأخرى.	21. felt that you were not free to do other daily life activities.
-	22. واجهت صعوبة في وضع خطة حياة شخصية.	22. had difficulty making personal life plan.
FB: Financial Burden (3 items)	32. لديك أي مشاكل مالية بسبب نفقات المريض الناجمة عن مرضه.	23. have any financial problems because of the expenses of patient’s disease.
-	42. فقدت وظيفتك لأنك تحتاج إلى مزيد من الوقت لرعاية مريضك المصاب بالفصام.	24. lost your job because you need more time to care for your patient with schizophrenia.
-	52. أجلت القيام بأنشطة أخرى مخخط لها مسبقا و تحتاج لتمويل.	25. postponed other planned activities that need funds.
